# Exploring the Roles of Liver X Receptors in Lipid Metabolism and Immunity in Atherosclerosis

**DOI:** 10.3390/biom15040579

**Published:** 2025-04-14

**Authors:** Kaori Endo-Umeda, Makoto Makishima

**Affiliations:** Division of Biochemistry, Department of Biomedical Sciences, Nihon University School of Medicine, 30-1 Oyaguchi-kamicho, Itabashi-ku, Tokyo 173-8610, Japan; makishima.makoto@nihon-u.ac.jp

**Keywords:** liver X receptor, cholesterol, inflammation, leukocyte, macrophage, atherosclerosis

## Abstract

Hypercholesterolemia causes atherosclerosis by inducing immune cell migration and chronic inflammation in arterial walls. Recent single-cell analyses reveal the presence of lipid-enriched foamy macrophages, as well as other macrophage subtypes, neutrophils, T cells, and B cells, in atherosclerotic plaques in both animal models and humans. These cells interact with each other and other cells, including non-immune cells such as endothelial cells and smooth muscle cells. They thereby regulate metabolic, inflammatory, phagocytic, and cell death processes, thus affecting the progression and stability of atherosclerotic plaques. The nuclear receptors liver X receptor (LXR)α and LXRβ are transcription factors that are activated by oxysterols and regulate lipid metabolism and immune responses. LXRs regulate cholesterol homeostasis by controlling cholesterol’s transport, absorption, synthesis, and breakdown in the liver and intestine. LXRs are also highly expressed in tissue-resident and monocyte-derived macrophages and other immune cells, including both myeloid cells and lymphocytes, and they regulate both innate and adaptive immune responses. Interestingly, LXRs have immunosuppressive and immunoregulatory functions that are cell-type-dependent. In animal models of atherosclerosis, LXRs have been shown to be involved in both progression and regression phases. The pharmacological activation of LXR enhances cholesterol efflux from macrophages and promotes atherosclerosis progression. Deleting LXR in immune cells, especially myeloid cells, accelerates atherosclerosis by increasing monocyte migration, macrophage proliferation and activation, and neutrophil extracellular traps (NETs); furthermore, the deletion of hematopoietic LXRs impairs the regression of atherosclerotic plaques. Therefore, LXRs in immune cells may be a potent therapeutic target for atherosclerosis.

## 1. Introduction

Atherosclerosis is a chronic disease induced by lipid accumulation and inflammation in arterial walls and is a major cause of cardiovascular disease. The dysfunction of endothelial cells in the arterial lumen triggers atherosclerosis by inducing the infiltration of low-density lipoproteins (LDLs) into the intima (the inner layer of arteries), where they are oxidized by reactive oxygen species; this promotes the recruitment of monocytes and their differentiation into macrophages, ultimately resulting in inflammatory responses [1]. Smooth muscle cells are transferred from the arterial media (between the intima and adventitia), and macrophages take up more lipids and transform into foam cells, resulting in the development of atherosclerotic plaques containing necrotic cores. These plaques develop into vulnerable plaques characterized by increased inflammation, a thin fibrous cap, and an enlarged necrotic core [2]. Recent reports reveal that both foam cells and other immune cells infiltrate in atherosclerotic plaques in both animal models and humans. Macrophages can be categorized into at least three types, each with a distinct role in atherosclerosis (refer to Section 2). Neutrophils accumulate in early atherosclerotic lesions and are activated by macrophages to induce neutrophil extracellular traps (NETs). Several types of lymphocytes are present in plaques. These findings highlight the increasing complexity of immune cell regulation in atherosclerosis progression.

The nuclear receptors liver X receptor (LXR)α (NR1H3) and LXRβ (NR1H2) were identified as orphan receptors in the 1990s [3,4], and they act as heterodimer partners of the retinoid X receptor (RXR), another nuclear receptor [5]. LXRα is mainly expressed in metabolic organs such as the liver, intestine, and kidneys, as well as macrophages, whereas LXRβ is present throughout the entire body [6]. LXRs are activated by oxysterols, which are cholesterol metabolites, such as 22(*R*)-hydroxycholesterol (22(*R*)-HC), 25-HC, 7α-HC, 24(*S*)-HC, and 27-HC [7,8,9]. The metabolites of 7-dehydrocholesterol, a cholesterol precursor in the Kandutsch–Russell pathway, can also activate LXRs [10]. These LXR-activating metabolites are produced by P450 enzymes such as sterol 27-hydroxylase, 24-hydroxylase, and cholesterol 25-hydroxylase (CH25H) [10,11]. Another natural ligand for LXRs is desmosterol, which is a precursor of cholesterol in the Bloch pathway produced by 24-dehydrocholesterol reductase (DHCR24) [12,13]. A liganded LXR forms a heterodimer with RXR and binds to two repeats of the nucleotide sequence AGGCTT. It is separated by four nucleotides in the same direction (called direct repeat 4) on the promoters of their target genes [14]. LXRs regulate the expression of various types of genes involved in lipid metabolism, immunity, cell proliferation, and apoptosis [15]. Numerous studies have demonstrated the antiatherogenic function of LXRs by assessing the effects of LXR activation and deletion in experimental models. In this review, we discuss recent findings relating to the antiatherogenic functions of LXRs in immune cells.

## 2. Immune Cells in Atherosclerotic Plaques

Single-cell RNA sequencing (scRNA-seq) and mass cytometry analyses have demonstrated that various types of immune cells infiltrate atherosclerotic plaques in animal models [16,17,18], in addition to human atherosclerotic plaques resected via endarterectomy [19,20,21,22]. Atherosclerotic lesions contain macrophage-derived foam cells, monocytes, inflammatory macrophages, neutrophils, dendritic cells (DCs), T cells, and B cells, which interact with each other and non-immune cells such as vascular endothelial cells and smooth muscle cells, contributing to the pathogenesis of atherosclerosis.

### 2.1. Monocytes and Macrophages

Monocyte-derived macrophages are the most important immune cells in both the progression and regression of atherosclerosis, as they play roles in lipid incorporation, inflammatory responses, and the phagocytosis and clearance of apoptotic cells in plaques. The circulating monocytes in mice are divided into the following two types: CD115 (macrophage colony-stimulating factor (M-CSF) receptor)-positive and high-Ly6C-expressing (Ly6C^hi^) monocytes; CD115-positive and low-Ly6C-expressing (Ly6C^lo^) monocytes. Feeding with a Western diet increased the number of Ly6C^hi^ monocytes, with high survival and proliferation activities observed in the circulation [23]. Experiments involving the labeling of Ly6C^hi^ monocytes with fluorescent latex beads showed that Ly6C^hi^ monocytes, rather than Ly6C^lo^ monocytes, accumulate in atherosclerotic plaques [24]. These findings indicate that Ly6C^hi^ monocytes are selectively recruited into the plaque lesions and are activated into macrophages. In addition, locally residing macrophages proliferate and contribute to the formation of atherosclerotic plaques [25]. Inflammatory macrophages derived from Ly6C^hi^ monocytes are differentiated after birth and in inflammatory conditions, while resident macrophages derive from hematopoietic progenitors before birth, and their pool is maintained via self-renewal [26]. Thus, in addition to monocyte-derived macrophages, tissue-resident macrophages are also present in atherosclerotic plaques. scRNA analysis in low-density-lipoprotein-receptor (LDLR)-deficient mice revealed that aortic macrophages could be divided into the following three types: resident-like macrophages, inflammatory macrophages, and triggering receptors expressed on myeloid cell 2 (TREM2)-expressing macrophages [16]. Additionally, monocytes with a higher expression of genes involved in cell proliferation and stem-cell-like signatures have been detected in both progression and regression stages in atherosclerotic lesions [27]. The arterial wall consists of three layers: the inner layer, called the intima, which contains endothelial cells; the middle layer, called the media, which contains vascular smooth muscle cells; and the outer layer, called the adventitia, which mainly contains fibroblasts [1]. In the steady state, arterial resident macrophages are highly present in the adventitia rather than the intima and media [26]. These cells highly express C-X3-C motif chemokine receptor 1 and have both embryonic and postnatal origins and self-renewal potential, but they do not have recruited monocyte characteristics [28]. While these resident macrophages are detected in the intima in the steady state, bone-marrow-derived monocytes differentiate into macrophages with similar characteristics in an M-CSF-dependent manner and proliferate in early atherosclerotic lesions [29]. Furthermore, the levels of inflammatory and TREM2^hi^ macrophages increase during the progression of atherogenesis, mainly being present in the arterial intima. Interestingly, the lipid-staining analysis of lesional macrophages using the fluorescent probe BODIPY493/503 revealed that the macrophages in the arterial intima (CD45/CD64/CD11b-positive cells) can be divided into the following two types: BODIPY-positive foamy macrophages and BODIPY-negative non-foamy macrophages [17]. The transcriptomes are markedly distinct between the following two types: non-foamy macrophages preferentially express pro-inflammatory genes such as *Il1b* (the gene encoding interleukin-1β; IL-1β), *Nlrp3* (the gene encoding nucleotide-binding oligomerization domain-like receptor family pyrin domain containing 3; NLRP3), and *Tnf* (the gene encoding tumor necrosis factor), whereas foamy macrophages express genes involved in lipid metabolism, such as *Nr1h3* (the gene encoding LXRα), *Abca1* (the gene encoding ATP-binding cassette (ABC) transporter A1), and *Cd36* (the gene encoding the scavenger receptor CD36). Interestingly, the *Trem2* gene is highly expressed in foamy non-inflammatory macrophages compared to non-foamy inflammatory macrophages [17,30]. TREM2 has been identified as a cell surface receptor associated with an adaptor protein called DNAX activation protein 12 [31]. It plays multiple roles in phagocytosis, lipid metabolism, cell survival, and inflammation in tissue-resident macrophages [32]. TREM2 is expressed in microglia [33] and regulates cholesterol metabolism [34]; its loss-of-function variant R47H has been correlated with the risk of Alzheimer’s disease [35,36]. It is also highly present in lipid-associated macrophages in adipose tissue [37] and the liver [38], as well as atherosclerotic plaques. TREM2 acts as a key factor for foamy macrophage formation and contributes to the stability of atherosclerotic plaques in animal models [30]. The deletion of TREM2 in macrophages decreased plaque sizes without affecting monocyte recruitment and inflammatory responses by impairing the proliferation and survival of foamy macrophages. TREM2 deficiency in hematopoietic cells increased the necrotic core size in early atherosclerosis, demonstrating the contribution of TREM2 in foamy macrophages to the clearance of dead cells (efferocytosis) [39]. Treatment with a TREM2 agonistic antibody increased the plaque area and macrophage accumulation, which is associated with the re-programming of foamy macrophage transcriptomes and improved plaque stability by inducing fibrous cap formation in smooth muscle cells, increasing collagen production and reducing the necrotic core area [40]. Another TREM2 agonist has also been shown to reduce necrotic core formation [39]. These findings indicate that TREM2 may be a key therapeutic factor for enhancing the functions of foamy macrophages, such as efferocytosis and plaque stability.

scRNA-seq analysis detected three or five distinct clusters of macrophages, which express the CD14, CD64, and/or CD68 myeloid cell marker genes, in human carotid atherosclerotic plaques (except the adventitia) [19,20,22]. Fernandez et al. identified the following five distinct clusters: three inflammatory macrophage clusters, one foamy macrophage cluster, and one unidentified macrophage cluster [19]. Macrophages in the three inflammatory clusters express distinct activation markers, including *HLA-DRA* (the gene encoding the major histocompatibility complex, class II, DR alpha) and *CD74* (the gene encoding the CD74 molecule); *CYBA* (the gene encoding the cytochrome b-245 alpha chain) and *LYZ* (the gene encoding the lysozyme); and *JUNB* (the gene encoding the JunB proto-oncogene and AP-1 transcription factor subunit) and *NFKBIA* (the gene encoding NFKB inhibitor alpha), respectively. Macrophages in the foamy cluster express genes involved in cholesterol metabolism, such as *APOE*. On the other hand, Wirka et al. reported the following three clusters: two inflammatory macrophage clusters and one foamy macrophage cluster [21]. Macrophages in the two inflammatory clusters highly express *IL1B* (the gene encoding IL-1β) and *CASP1* (the gene encoding caspase 1), and *TNF* (the gene encoding tumor necrosis factor) and *TLR4* (the gene encoding Toll-like receptor 4; TLR4), respectively. Macrophages in the foamy cluster express genes involved in lipid metabolism, such as *ABCA1* and *TREM2*. Furthermore, Bashore et al. initially identified five macrophage clusters, and a subsequent subclustering analysis revealed seven distinct clusters [22]. The subpopulations include two inflammatory macrophage clusters, two foamy macrophage clusters, one apoptotic macrophage cluster, one proliferative macrophage cluster, and one macrophage cluster with the higher expression of smooth-muscle-cell-related genes. An integrated analysis has been performed with two scRNA-seq datasets [41]. This analysis identified three major clusters, including inflammatory macrophages, foamy macrophages, and resident-like macrophages, and two minor clusters that highly express C3 (the gene encoding complement component 3) and JUN (the gene encoding the Jun proto-oncogene and AP-1 transcription factor subunit) and type-I interferon (IFN)-related genes. An integrated analysis of macrophages has been further performed with murine and human datasets, and it showed that the gene expression patterns of major human macrophage clusters are similar to those of murine clusters.

CD14-positive monocytes are present in both peripheral blood and atherosclerotic plaques [19]. An analysis involving the cellular indexing of transcriptomes and epitopes via sequencing (CITE-seq) and scRNA-seq revealed that circulating monocytes can be separated into eight clusters, including five classical monocytes, one non-classical monocyte, an IFN-responsive monocyte, and a monocyte with a higher expression of major histocompatibility complex (MHC) class II [42]. Among the clusters, the subset of classical monocytes, IFN-responsive monocytes, monocytes with a higher expression of MHC class II, and non-classical monocytes was differentially associated with cardiovascular disease risk factors such as race, smoking, and plasma LDL-cholesterol levels. A similar analysis detected various types of monocyte clusters as follows: eight classical (CD14-positive and CD16-negative), two non-classical (CD14-negative, CD16-positive, and CD56-negative), and five intermediate (CD14-positive and CD16-positive) monocytes [43]. Therefore, various types of monocytes and macrophages contribute to the pathogenesis of atherosclerosis, exhibiting cell-selective functions in both animal models and humans.

### 2.2. Neutrophils

In addition to monocytes and macrophages, neutrophils have been detected in arterial plaques in animal models. Hypercholesterolemia induced neutrophilia and neutrophil accumulation in an early stage of atherosclerosis in apolipoprotein E (Apo E)-deficient mice [44]. Additionally, neutrophils significantly infiltrated together with C-X3-C motif chemokine receptor 1-positive monocytes/macrophages, suggesting a pro-inflammatory phenotype [45]. Consistent with these findings, the antimicrobial peptide cathelicidin—secreted via activated neutrophils—promotes the adhesion of classical monocytes and increases atherosclerosis [46]. Additionally, lesional neutrophils undergo NET formation (NETosis) in plaques [47]. NETosis is induced through the production of reactive oxygen species via nicotinamide adenine dinucleotide phosphate hydrogen (NADPH) oxidase and histone citrullination and chromatin decondensation via peptidylarginine deiminase (PAD)4, which is highly expressed in neutrophils. Treatment with the PAD inhibitor Cl-amidine suppressed atherosclerosis and arterial thrombosis in Apo E-null mice [47]. The inhibition of NETosis through the deletion of neutrophil-specific protease and elastase and treatment with the NADPH oxidase inhibitor diphenylene iodonium or deoxyribonuclease I reduced atherosclerosis, indicating that NETosis functions as a pro-atherogenic factor [48]. Interestingly, NETs directly activate macrophages to produce pro-inflammatory cytokines. A lack of PAD4 in hematopoietic cells suppressed NETosis in the plaque but did not reduce the size and stability of the plaque in LDLR-deficient mice [49]. Similarly, NETosis was still detected in PAD4-knockout plaques [50]. These findings suggest that histone citrullination via PAD4 is not necessary for NETosis but enhances NET-mediated inflammation. Neutrophil infiltration has also been detected in human specimens with myocardial infarction [51] and atherosclerotic plaques in carotid arteries, associated with the expression of NADPH oxidase subunit [52] and NETosis [49]. Thus, neutrophils act as atherogenic factors by promoting NETosis and enhancing macrophage inflammation in both animal models and humans.

### 2.3. Dendritic Cells

DCs are also present in atherosclerotic plaques and influence the progression of atherosclerosis. DCs are professional antigen-presenting cells from common hematopoietic stem-cell-derived DC precursors. Mature DCs are divided into type 1 conventional DCs (cDC1), type 2 conventional DCs (cDC2), and plasmacytoid DCs (pDC) [53]. The development of these subsets is regulated by distinct transcription factors. A cDC1 subset is regulated by the transcription factor basic leucine zipper transcriptional factor ATF-like 3; a cDC2 subset is regulated by interferon-regulatory factor 4; a pDC subset is regulated by E2-2. Among these subsets, the pDC subset is present in mouse atherosclerotic lesions [54,55,56]. Their depletion via antibody treatments or genetic manipulation prevents atherosclerosis. pDCs are also detected in human atherosclerosis and produce IFN-α, which is associated with plaque vulnerability [57]. Thus, pDCs promote the progression of atherosclerosis.

### 2.4. Lymphocytes

Both innate and adaptive immunity types are involved in the progression of atherosclerosis. scRNA-seq and mass cytometric analyses have demonstrated that several types of T cells, including CD4-positive Th2 cells and Th17 cells, CD8-positive T cells, C-X-C motif chemokine ligand 6-positive T cells, and memory T cells, are present in atherosclerotic plaques in animal models [16,18]. Each T cell subset has a distinct role as follows: Th1 cells are pro-atherogenic, while regulatory T cells are antiatherogenic, although the roles of Th2-positive, Th17-positive, and CD8-positive T cells remain controversial [58]. CD4-positive, CD8-positive, and regulatory T cells are clonally expanded and dysregulated in advanced plaques, suggesting that the local conditions, such as lipid accumulation and inflammation, induce the production of autoantigens to activate these T cells [59]. CD4-positive T cells and CD8-positive T cells have also been shown to be present in human atherosclerotic plaques [19]. In humans, T cells are predominantly present in greater numbers than monocytes/macrophages compared to murine atherosclerosis [60]. scRNA-seq analyses revealed that CD4-positive T cells are activated and upregulate the IFN-γ, IL-1, and IL-6 signaling pathways along with macrophages [19]. Some populations of CD8-positive T cells exhibit a higher expression of T cell exhaustion markers. Natural killer (NK) T cells—a subset of T cells that are restricted by MHC class I-like molecule CD1d and express both conventional T cell and NK cell surface markers—are also present in human carotid plaques. The removal of NKT cells in LDLR-deficient or Apo E-deficient mice via CD1d gene deletion protected against the progression of atherosclerosis, demonstrating that NKT cells promote atherogenesis [61,62].

B cells are another important subset of lymphocytes. Murine B cells are divided into two subsets, B1 and B2 cells, with distinct cell surface markers and functions, and both B cells are present in atherosclerotic plaques [18,63]. B1 cells protect against atherosclerosis by producing antibodies against oxidized LDL and inhibiting the lipid uptake and inflammatory functions of macrophages. On the other hand, the depletion of B2 cells inhibits T cell proliferation and cytokine production, resulting in reduced atherosclerosis [64]. Thus, the effects of B cells in atherosclerosis are subset-selective; B1 cells are atheroprotective, while B2 cells promote atherogenesis.

## 3. LXRs Regulate Lipid Metabolism and Immune Function

### 3.1. Regulation of Lipid Metabolism

**Figure 1:**
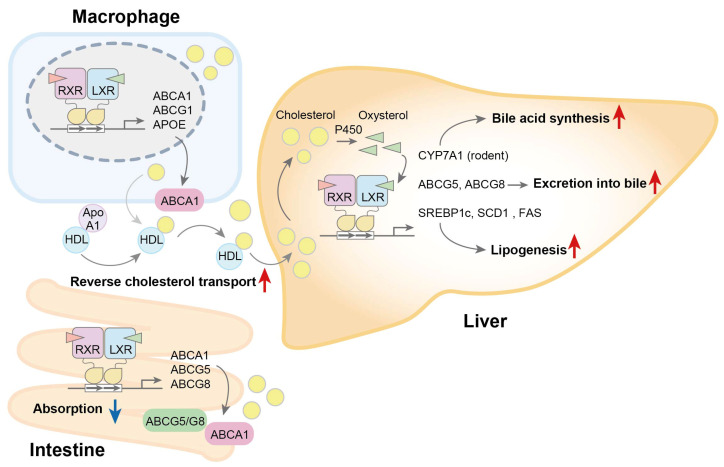
LXRs regulate cholesterol metabolism in multiple tissues. In peripheral tissues, including macrophages, excess cholesterol results in increased oxysterol levels, which activate LXRs, thereby upregulating the expression of the ABC transporters ABCA1 and ABCG1 and promoting reverse cholesterol transport. Hepatic LXR activation stimulates the conversion of cholesterol to bile acids by inducing cholesterol 7α-hydroxylase (CYP7A1), but this occurs only in rodents. It also elevates the expression of ABCG5 and ABCG8, stimulating cholesterol excretion into bile in both rodents and humans. Furthermore, LXR activation promotes de novo lipogenesis by inducing the expression of lipogenic genes such as sterol regulatory element-binding protein-1c (SREBP1c), stearoyl-CoA desaturase-1 (SCD1), and fatty acid synthase (FAS). In the intestine, LXR activation upregulates ABC transporters and inhibits cholesterol absorption. Red arrows, up; blue arrows, down.

Hepatic LXRs increase the expression of ABCG5 and ABCG8 to induce the excretion of cholesterol into bile [65] (Figure 1). In a rodent liver model, LXRα promoted bile acid synthesis by upregulating the expression of cholesterol 7α-hydroxylase [66]. LXRα-deficient but not LXRβ-deficient mice fed a Western diet containing high cholesterol presented marked hepatic cholesterol accumulation [67] and severe liver injury [68]. LXRs also regulate LDLR protein stability by inducing the gene of myosin regulatory light chain interacting protein (MYLIP, also called the inducible degrader of the LDLR, i.e., IDOL), which acts as an E3 ubiquitin ligase against LDLR proteins [69]. The ectopic expression of *MYLIP* in the liver strongly reduces LDLR expression and accelerates atherosclerosis [70]. The lower expression of *ABCA1*, *ABCG1*, and *MYLIP* has been associated with a risk of pre-diabetes and type 2 diabetes in humans [71]. In addition to the regulation of cholesterol metabolism, LXRs stimulate de novo lipogenesis by inducing the expression of the genes of sterol regulatory element-binding protein-1c [72,73], stearoyl-CoA desaturase-1 [72], and fatty acid synthase [74]. The pharmacological activation of LXRs in mice increased the expression of these lipogenic genes in the liver and induced hypertriglyceridemia [75]. Intestinal LXRs induced the expression of the ABC transporter genes *ABCA1*, *ABCG5*, and *ABCG8*, and they suppressed dietary cholesterol absorption [65,76]. In macrophages, LXRs have been shown to upregulate *ABCA1* [77], *ABCG1,* and *APOE* [78] and to stimulate cholesterol reverse transport. The administration of an LXR agonist promoted cholesterol reverse transport from macrophages in vivo [79]. Thus, as metabolic sensors for oxysterols, LXRs regulate lipid metabolism.

### 3.2. Regulation of Immune Response

**Figure 2:**
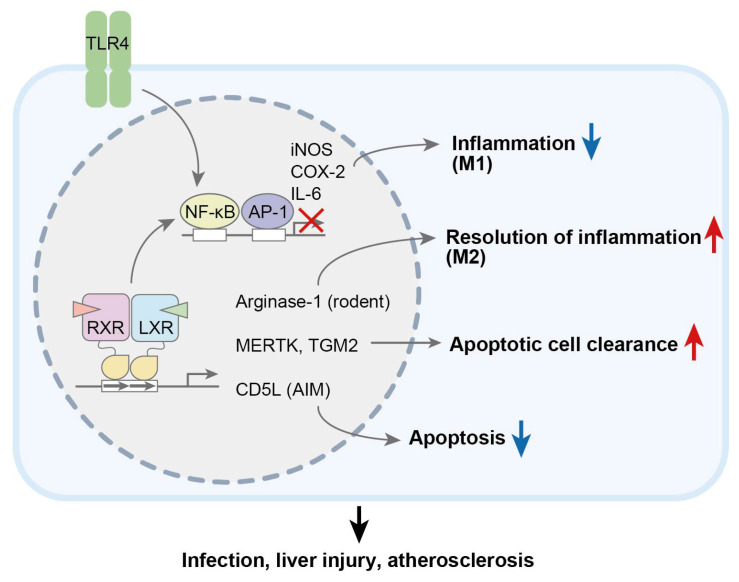
Multiple roles of LXRs in macrophage functions. In inflammatory macrophages (M1 macrophages), LXR activation suppresses the expression of pro-inflammatory cytokines directly or indirectly through NF-κB repression. In contrast, LXR activation increases arginase-1 expression and promotes the resolution of inflammation in anti-inflammatory M2 macrophages, although arginase-1 is not induced in human macrophages. LXRs also induce the expression of MERTK and TGM2, promoting the clearance of apoptotic cells, a process known as efferocytosis. Furthermore, LXRs suppress macrophage apoptosis by inducing CD5L (also known as AIM). Thus, LXRs influence the pathogenesis of infection, liver injury, and atherosclerosis by modulating macrophage function. Red arrows, up; blue arrows, down.

LXRs play important roles in both innate and adaptive immunity. LXR ligand treatment inhibited the expression of pro-inflammatory genes, such as the genes for nitric oxide synthase, cyclooxygenase-2, and IL-6, which are induced by TLR ligands in primary macrophages [80] (Figure 2). LXRs repressed nuclear factor-κB (NF-κB) transcription activity through small ubiquitin-like modifier (SUMO)ylation [81,82,83], induced the cell membrane ABCA1/caveolin-1-mediated suppression of inflammation [84,85], and directly inhibited the expression of inflammatory genes by modulating their chromatin accessibilities [86]. Additionally, LXR activation inhibits macrophage proliferation by regulating the expression levels of cyclins and cyclin-dependent kinases [87].

LXRα and LXRβ have been shown to commonly and distinctly regulate gene expression in several biological processes in mouse macrophage RAW264.1 cells and primary macrophages, such as bone-marrow-derived macrophages and peritoneal macrophages [88]. LXRs also play roles in the development and function of tissue-resident macrophages. LXRα is highly expressed in splenic red-pulp macrophages and liver-resident Kupffer cells [89]. LXRα-deficient mice exhibited the defective differentiation of splenic marginal zone macrophages [90] and hepatic Kupffer cells [91,92]. An increase in the number of hematopoietic stem cells and myeloid progenitor cells was observed in LXR-deficient mice [93], as well as in hypercholesterolemic Apo E-deficient mice [94]. Bone-marrow-derived inflammatory macrophages increase to compensate for the defects of tissue macrophages, such as hepatic Kupffer cells [95]. This is a phenotype that has been suggested to render LXR-deficient mice susceptible to lipopolysaccharide-induced liver injury [95], *Listeria monocytogenes* infection [96], and metabolic-dysfunction-associated steatohepatitis [68]. On the other hand, LXR ligand treatment directly induced TLR4 expression, inflammatory responses, and the production of reactive oxygen species in human macrophages [97]. The long-term stimulation of human macrophages with an LXR agonist enhances the pro-inflammatory response induced by lipopolysaccharides, whereas short-term LXR activation suppresses it. LXR activation enhanced the expression of IL-1β, and some glycolytic genes with activating hypoxia-induced factor 1α [98,99] inhibited the anti-inflammatory macrophage polarization mediated by M-CSF and potentiated the pro-inflammatory phenotype in humans [100]. LXR activation also induces pro-inflammatory innately trained immunity in human monocytes through epigenetic and metabolic reprogramming [101]. These findings suggest that the role of LXR signaling in macrophages differs between humans and mice. Arginine metabolism is regulated by inducible NO synthase (iNOS) and arginase-1, and it influences macrophage function. In mice, pro-inflammatory macrophages, called M1, express iNOS, which generates nitric oxide and induces inflammatory responses, whereas anti-inflammatory macrophages, called M2, express arginase-1, which decreases arginine and promotes the resolution of inflammation. However, human macrophages fail to induce arginase-1 expression under M2 conditions. The difference in arginine metabolism may contribute to the distinct LXR responses between human and mouse macrophages [102]. Interestingly, LXR activation induces the expression of transglutaminase 2 (TGM2) in both human and mouse macrophages (discussed in Section 3.3), which is involved in anti-inflammatory efferocytosis. Further investigations are required to determine whether the differences in the roles of LXR are due to species-related differences or differences in macrophage types.

LXRs also influence the function of other types of myeloid cells, such as DCs [103,104,105], mast cells [106], and neutrophils [107]. LXRα is highly expressed in human-monocyte-derived DCs, and its activation suppresses DC maturation and the ability to activate T cells [103]. In contrast, LXR activation promotes DC maturation marker expression and chemotaxis [104,105]. Thus, the role of LXRs in monocyte-derived DCs is controversial. Recently, it has been reported that LXRβ rather than LXRα plays an important role in regulating the tolerogenic or immunogenic maturation of cDC1 in vivo [108]. LXRβ also plays an anti-inflammatory role in human pDCs by inducing the efferocytic receptor BAI-1 [109]. The anti-inflammatory effect of LXR activation is also observed for human pDC cells that exhibit interference with NF-κB induction [110]. In addition to myeloid cells, LXRs affect the functions of lymphocytes, including T cell proliferation [111], thymic differentiation, and the maturation of T cells and NKT cells [112,113]. LXR signaling also controls T cell differentiation, such as in IL-17-producing Th17 cells [114,115], follicular helper CD4-positive T cells [116], and regulatory T cells [117]. Furthermore, LXRs regulate immunoglobulin E production in B cells [118]. LXR deletion and hypercholesterolemia accelerate autoimmune disease through the activation of CD11c-positive antigen-presenting cells, thus promoting T cell priming and increasing B cell expansion [119]. These findings reveal that LXRs influence the differentiation and function of both tissue-resident and bone-marrow-derived immune cells. Interestingly, TLR signaling activation suppressed LXR target gene expression in macrophages [120], suggesting that LXRs serve as a hub between lipid metabolism and the innate immune response.

### 3.3. Phagocytosis and Apoptosis

LXRs have been shown to contribute to apoptotic cell clearance by inducing *Mertk*, the gene for MER tyrosine kinase (MERTK), which is involved in phagocytosis, in mouse primary macrophages [121] and tissue-resident macrophages in the liver and spleen [122] (Figure 2). LXR-deleted mice presented defective phagocytosis and the increased production of autoantibodies against nuclear proteins, and they exhibited systemic autoimmune disease. *Mertk*-deficient mice presented accelerated atherosclerosis [123], and the expression of LXRα was induced by a transcription factor activating transcription factor 6 during efferocytosis in macrophages [124]. Thus, the LXR-MERTK pathway plays an important role in the suppression of atherosclerosis through the induction of efferocytosis in atherosclerotic plaques. TGM2 also participates in apoptotic cell removal and exerts anti-inflammatory effects. LXR activation induces the expression of the *TGM2* gene and promotes macrophage phagocytosis in both humans and mice [125,126]. Mechanistically, the activation of LXR-RXR directly upregulates the expression of nuclear receptor retinoic acid receptor (RAR)α, and then, TGM2 is consequently upregulated via RARα-RXR activation. TGM2 and RARα are expressed in monocytes isolated from human atherosclerotic plaques [125], suggesting that this pathway is activated to suppress atherosclerosis. On the other hand, hypercholesterolemia and LXR activation also induce CD5L (or apoptosis inhibitors expressed by macrophages, i.e., AIM) and suppress the apoptosis of macrophages [127]. CD5L-deficient mice showed reduced atherosclerosis due to the promotion of macrophage apoptosis in lesions. The expression of CD5L is reciprocally regulated by LXR and the transcription factor MafB [128]. The deletion of MafB accelerated foam cell apoptosis and ameliorated atherosclerosis. *LBP*—the gene for lipopolysaccharide-binding protein (LBP)—is a target gene of LXR [129]. LBP-null mice also presented reduced atherosclerosis due to increased foam cell apoptosis. These findings indicate that macrophages contribute to the pathogenesis of atherosclerosis through foam cell formation, but they suppress atherosclerosis via efferocytosis to eliminate apoptotic cells. The antiatherogenic functions of LXRs have been suggested to be mediated by enhanced efferocytosis functions and survival in a specific subtype of macrophages.

## 4. Effect of Cholesterol Accumulation in Atherosclerotic Plaques

A Western diet induces systemic inflammation by reprogramming myeloid cells [130]. Cholesterol accumulation is the most important factor for the induction of inflammation in macrophages and other immune cells, as well as for the progression of atherosclerosis [131].

The genes for ABC transporters *ABCA1* and *ABCG1* are LXR targets and ABCA1 and ABCG1 strongly influence atherosclerosis both LXR-dependently and LXR-independently. Both transporters are selectively expressed in foamy macrophages rather than non-foamy inflammatory macrophages [17]. The deletions of ABCA1 and ABCG1 in primary macrophages increase the expression of Toll-like receptors, followed by that of pro-inflammatory cytokines and chemokines [132]. The bone marrow transplantation (BMT) of myeloid cell-specific ABCA1/G1-deficient cells induced monocytosis and neutrophilia and accelerated atherosclerosis, with an enlarged necrotic core area observed [133]. The non-coding RNA *MeXis* was induced by LXRs and cooperatively regulated *Abca1* expression with LXRs [134]. The deletion of *MeXis* resulted in defects in cholesterol efflux and enhanced atherogenesis. Cholesterol crystals increased in atherosclerotic plaques, directly activating the NLRP3 inflammasome pathway as a damage-associated molecular pattern and resulting in IL-1β production [135]. The absence of NLRP3 and the adaptor protein ASC or IL-1α/β in bone marrow cells drastically reduces atherosclerosis, indicating that cholesterol-induced inflammasome activation plays an important role in atherogenesis. The Canakinumab Anti-inflammatory Thrombosis Outcome Study revealed that the use of a blocking antibody against IL-1β was able to significantly reduce the future risks of heart attacks and strokes without affecting plasma lipid levels [136]. Consistent with these observations, cholesterol accumulation via myeloid cell ABCA1/G1 deletion resulted in the activation of the NLRP3 inflammasome pathway, increased IL-1β and IL-18 production, and increased atherosclerotic progression [137]. Interestingly, the cholesterol accumulation and IL-1β production caused by inflammasome activation in macrophages, but not in neutrophils, triggered the induction of NETosis in plaques [138]. These findings indicate that excess cholesterol accumulation initiates NLRP3 inflammasome activation, which is the most important process for the promotion of atherosclerosis, and this suggests that the initiation of atherosclerosis is controlled by LXRs as cholesterol metabolism sensors. Studies regarding the effects of LXR activation and deletion on atherosclerosis in animal models are summarized in Table 1, Table 2 and Table 3, and they are discussed below.

## 5. Pharmacological and Endogenous LXR Activation in Animal Models of Atherosclerosis

### 5.1. Pharmacological LXR Activation

Treatment with the synthetic LXR agonist GW3965 ameliorates atherosclerosis in LDLR-deficient and Apo E-deficient mice [139] (Table 1). LDLR-null mice, but not Apo E-null mice, exhibited reduced total plasma cholesterol levels after 12 weeks of Western diet treatment. Another LXR ligand, T0901317, also reduced atherosclerosis in LDLR-deficient mice without altering total plasma cholesterol levels [140]. The pharmacological activation of LXR exerts antithrombotic [141] and antiatherosclerotic effects by inhibiting apoptosis, enhancing the efferocytosis of lesional macrophages, and suppressing endoplasmic reticulum stress [142]. T0901317 administration also improved the regression of atherosclerosis and plaque stability in rheumatoid arthritic mice [143]. Interestingly, T0901317 or GW3965 administration still suppressed plasma C-C chemokine 2 levels and reduced atherogenesis in LDLR-null mice transplanted with bone marrow cells isolated from myeloid-specific ABCA1/G1-deficient mice [144]. These findings indicate that myeloid cell LXRs play a role independent of the cholesterol efflux pathway in preventing atherogenesis. GW3965 promoted the differentiation of endothelial progenitor cells into an anti-arteriosclerotic phenotype by decreasing the expression of the endothelial lineage markers *Cd144* and *Vegfr2* and inhibiting adhesion to monocytes [145]. The administration of GW3965-treated endothelial progenitor cells to LDLR-deficient mice suppressed atherosclerotic lesions, with a decreased expression of vascular cell adhesion molecule 1. As treatment with conditioned media from GW3965-treated endothelial progenitor cells inhibits monocyte adhesion, secreted factors are considered to be involved in the atheroprotective function. Although LXR ligand activation stimulated cholesterol efflux and attenuated atherosclerosis, it induced hepatic de novo lipogenesis followed by hypertriglyceridemia [75]. To prevent this adverse effect, nanocarriers encapsulated with the agonist were synthesized to target macrophage LXRs [146,147]. These modified ligands effectively increased ABCA1 and ABCG1 expression in macrophages and suppressed atherosclerotic progression without inducing hepatic lipogenic gene expression. Cotreatment with T0901317 and the AMP-activated protein kinase activator metformin synergistically protected against atherosclerosis and improved plaque stability in Apo E-deficient mice [148]. In this model, metformin enhances T0901317-induced cholesterol transport and inflammation suppression, and it can also counteract hepatic lipogenesis. In addition, the mitogen-activated protein kinase inhibitor U0126 exhibited a synergistic antiatherogenic effect with T0901317 without inducing lipogenic gene expression [149,150], and cotreatment with T0901317 and reconstituted high-density lipoproteins (rHDLs) cooperatively reduced atherosclerosis progression [151]. Although treatment with rHDL induced both anti-inflammatory and pro-inflammatory effects in a cholesterol-dependent manner in bone-marrow-derived macrophages, its administration suppressed inflammatory gene expression in lesional CD11b-positive myeloid cells in Apo E-null mice, suggesting its therapeutic potency in vivo [152].

**Table 1:** Effects of liver X receptor (LXR) activation in animal models of atherosclerosis.

Mice	Model	LXR	Diet	Treatment	Findings	Ref
LDLR(-/-) and Apo E(-/-)	Progression	Pharmacological activation	WTD (12 weeks)	GW3965 (1 or 10 mpk)	Lesion size↓ ABCA1, ABCG1↑ (aorta)	[139]
LDLR(-/-)	Progression	Pharmacological activation	WTD (8 weeks)	T0901317 (3 or 10 mpk)	Lesion size↓ ABCA1, ABCG1↑ (plaque)	[140]
Apo E(-/-)	Progression	Pharmacological activation	WTD (4 weeks)	GW3965 (10 mpk)	Lesion size↓ Macrophage apoptosis↓, efferocytosis↑ Endoplasmic reticulum stress↓	[142]
LDLR(-/-)	Regression	Pharmacological activation	WTD (14 weeks) →ND (3 weeks)	T0901317 (25 mpk) and/or K/BxN serum with ND	Plaque regression and stability↑ Arthritic clinical score↓ Inflammation↓	[143]
LDLR(-/-)	Progression	Pharmacological activation	WTD (12 weeks)	BMT (myeloid ABCA1/G1(-/-)) T0901317 or GW3965 (10 mpk)	Lesion size↓ Immune cells↓ (aorta) Plasma chemokine↓	[144]
LDLR(-/-)	Progression	Pharmacological activation	WTD (8 weeks)	GW3965-treated endothelial progenitor cells	Lesion size↓ Expression of vascular cell adhesion molecule 1↓	[145]
Apo E(-/-)	Regression	Pharmacological activation by nanoparticle-mediated delivery	WTD (14 weeks) →ND (6 weeks)	T0901317 (1.5 mpk) in synthetic HDL nanoparticles (30 mpk)	Lesion size↓ Hepatic lipogenic gene, plasma TG→	[146]
Apo E(-/-)	Progression	Pharmacological activation by nanoparticle-mediated delivery	WTD (16 weeks)	T0901317 (5 mpk) in nanofiber hydrogel (0.2%)	Lesion size↓ Hepatic lipogenic gene→	[147]
Apo E(-/-)	Progression	Pharmacological activation	WTD (16 weeks)	T0901317 (1 mpk) and/or metformin (100 mpk)	Lesion size: T0901317↓, T0901317 + metformin↓ Plaque stability↑ Hepatic lipogenesis→	[148]
Apo E(-/-) or LDLR(-/-)	Progression	Pharmacological activation	WTD (16 or 20 weeks)	T0901317 (1 mpk) and/or U0126 (3 mpk)	Lesion size↓(T0901317) ↓(T0901317 + U0126) Hepatic lipogenesis→	[149,150]
Apo E(-/-)	Progression	Pharmacological activation	WTD (13 weeks)	T0901317 (1.5 mpk) and/or rHDL (30 mpk)	Lesion size↓ (T0901317 + reconstituted HDL) Cholesterol efflux↑	[151]
Apo E(-/-)	Progression	Pharmacological activation	WTD (11 weeks)	*N,N*-dimethyl-3β-hydroxy-cholenamide (8 mpk)	Lesion size↓ Macrophage infiltration↓ Hepatic lipogenic gene→	[153]
Apo E(-/-)	Progression	Pharmacological activation	WTD (12 weeks)	Nagilactone B (10 and 30 mpk)	Lesion size↓ ABCA1↑ (plaque macrophage) Hepatic lipogenic gene→	[154]
LDLR(-/-)	Progression	Pharmacological activation	WTD (8 weeks)	WAY-252623 (LXR-623) (15 mpk)	Lesion size↓ Hepatic lipogenic gene→	[155]
Apo E(-/-); Lipid droplet-associated hydrolase-transgenic	Progression	Endogenous activation	WTD (20 weeks)		Lesion size↓ LXR target genes↑	[156]
LDLR(-/-)	Progression	Endogenous activation	WTD (13 weeks)	SH42 (0.5 mpk)	Desmosterol levels↑ Lesion size→	[157]
LDLR(-/-)	Progression	Liver-specific overexpression	WTD (12 weeks)	Adeno-associated virus gene transfer of LXRα	Lesion size↓ LXR target genes↑	[158]
LDLR(-/-); Intestinal-specific VP16-LXRα transgenic	Progression	Intestine-specific overexpression	WTD (16 weeks)		Lesion size↓ Cholesterol absorption↓ Reverse cholesterol transport↑	[159]

ND, Normal diet; WTD, a Western diet high in fat and cholesterol; BMT, bone marrow transplantation; rHDL, reconstituted HDL. Arrows indicate the following: ↑, increase; ↓, decrease; →, no change.

Other various LXR modulators with isoform-selective or function-selective features have been synthesized as therapeutic candidates for LXR-related diseases, including atherosclerosis [160,161,162,163]. The synthetic steroid *N,N*-dimethyl-3β-hydroxy-cholenamide and the plant-derived compound nagilactone B are LXR agonists, which can reduce atherosclerosis without hepatic lipogenic gene induction [153,154]. The selective LXR synthetic modulator WAY-252623 (LXR-623) reduced the atherosclerotic lesion size in LDLR-null mice but exhibited few lipogenic effects in hamsters and significantly decreased serum LDL-cholesterol levels in non-human primates [155]. A clinical trial in healthy volunteers showed that this ligand induces ABCA1 and ABCG1 expression, but it exhibits central-nervous-system-related adverse effects [164]. The LXRβ-selective ligand BMS-852927 increased the expression of LXR target genes in blood cells, but it also elevated plasma LDL-cholesterol and triglycerides while decreasing high-density lipoprotein (HDL) cholesterol and circulating neutrophils in human subjects [165]. Therefore, pharmacological LXR activation exhibits protective effects against atherosclerosis by promoting cholesterol efflux and the inhibition of inflammation. Recently, phase I clinical trials of the LXR agonist abequolixron (RGX-104) have been conducted in subjects with advanced lung and endometrial cancer or non-small cell lung cancer (ClinicalTrials.gov ID NCT02922764, NCT05911308). The trials have been terminated, but the results have not been reported. Further investigations are needed to develop function-selective ligands without adverse effects.

### 5.2. Endogenous LXR Ligands: Oxysterols

LXR-activating oxysterols can also suppress atherosclerosis. Oxysterol levels are regulated through their synthesis and storage. Lipid-droplet-associated hydrolase, a protein present in lipid droplets, is expressed in macrophages in plaques and acts as an antiatherogenic factor [156]. This enzyme reduces sterol ester storage and increases non-esterified forms of oxysterols, which activate LXRs and induce anti-inflammatory responses.

Oxysterols differentially influence the pathogenesis of atherosclerosis in a hydroxy-group position-dependent manner. Desmosterol is elevated in macrophages in response to excess cholesterol and acts as an endogenous LXR ligand in addition to 25-HC and 27-HC [13]. The depletion of desmosterol through the overexpression of hepatic DHCR24 increased atherosclerosis plaque size and necrotic core formation by inducing inflammasome activation in lesional macrophages [166]. However, while treatment with the DHCR24 inhibitor SH42 increased desmosterol levels and decreased non-classical blood monocytes. It did not affect the development of atherosclerosis; this was probably due to an insufficient increase in desmosterol for LXR activation in macrophages [157]. The deletion of CH25H accelerated atherosclerotic lesion formation and macrophage accumulation, suggesting that 25-HC exhibits an atheroprotective function via LXR activation [167]. The expression of both LXRα and CH25H is regulated by the transcription factor Krüppel-Like Factor 4 in endothelial cells and macrophages. In contrast, the BMT of CH25H-deficient cells improved the lesion area, necrotic core, lesion inflammation, and plaque instability [168]. 25-HC is present in coronary atherosclerotic plaques in humans, which is associated with higher expression of the CH25H gene. These findings suggest that 25-HC has an LXR-independent pro-atherogenic effect. Elevated 27-HC levels in macrophages induced by the genetic deletion of its producing enzyme accelerated atherosclerosis due to the production of pro-inflammatory cytokines and leukocyte–endothelial cell adhesion in an estrogen receptor α-dependent but LXR-independent manner [169,170]. Therefore, oxysterols play dual roles in both suppressing and promoting atherosclerosis, modulating the functions of endothelial cells and macrophages.

## 6. LXR Deletion in Mouse Models of Atherosclerosis

The systemic deletion of LXRα but not LXRβ increased hepatic cholesterol accumulation [67]. LXRα-/β-deficient mice exhibited more severe lipid accumulation in the aorta compared to LXR single-knockout mice [171]. LXRα deletion enhanced cholesterol accumulation in peripheral tissues, impaired cholesterol reverse transport, and accelerated atherosclerosis in Apo E-deficient mice [172] (Table 2). The administration of a synthetic LXR agonist in mice deficient in both LXRα and Apo E could revert whole-body cholesterol levels and the formation of atherosclerotic plaques, indicating that LXRβ—in addition to LXRα—has an atheroprotective function. The non-redundant roles of LXRα and LXRβ have also been observed in LDLR-deficient mice [173]. Consistent with these findings, LXRα/LXRβ/Apo E triple-knockout mice exhibited significantly more severe cholesterol accumulation and were not able to survive over 10 weeks [174]. Interestingly, the atherosclerotic lesion in LXRβ/Apo E double-knockout mice was comparable with that in Apo E single-knockout mice, indicating the supportive role of LXRβ.

The hepatic expression of ABCG5 and ABCG8 is selectively regulated by LXRα and not LXRβ. Inducing the hepatocyte-specific expression of LXRα through adeno-associated virus gene transfer resulted in a reduced atherosclerotic lesion area [158] (Table 1), and its deletion in hepatocytes impaired cholesterol excretion and accelerated atherosclerosis [175] (Table 2). The hepatic overexpression of sulfotransferase family cytosolic 2B member 1 reduced oxysterol production, inhibited LXR activation and repressed reverse cholesterol transport under a high-cholesterol diet [176]. Therefore, hepatic LXRα is involved in reverse cholesterol transport throughout the entire body, in addition to metabolism in the liver, and it contributes to atheroprotection to a greater extent than LXRβ, which plays a limited role in hepatocytes. The intestine-selective activation of LXRα also reduced cholesterol absorption, promoted cholesterol reverse transport, and ameliorated atherosclerosis [159] (Table 1). Thus, hepatic and intestinal LXRα play leading roles in protecting against atherosclerosis and regulating whole-body cholesterol homeostasis.

**Figure 3:**
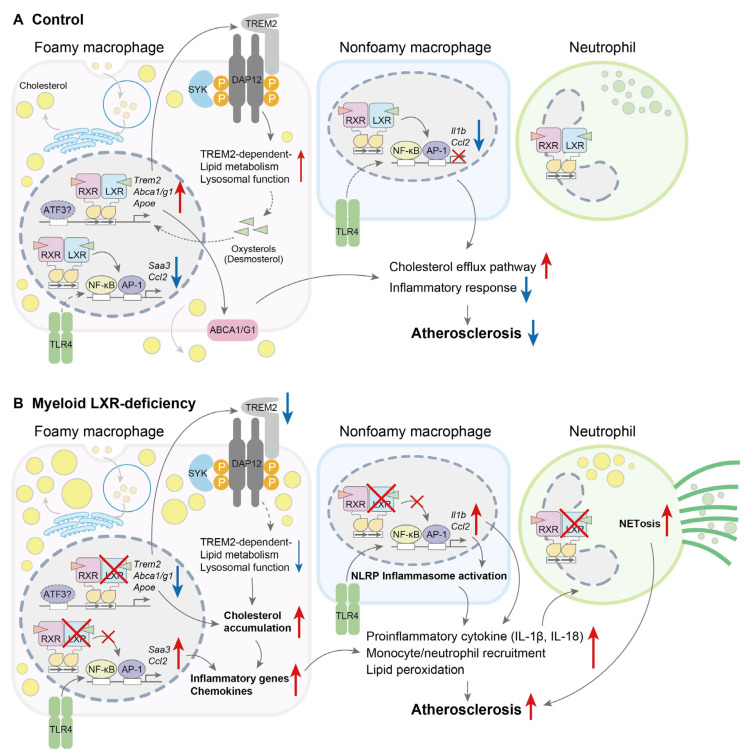
Effects of myeloid liver X receptor (LXR)α/β deficiency in mouse models of atherosclerosis. (**A**) In control mice, LXRs are activated via increased cholesterol levels and promote the cholesterol efflux pathway in non-inflammatory foamy macrophages. LXRs also repress inflammatory responses, such as the inflammasome pathway, in non-foamy inflammatory macrophages. As a consequence, neutrophil recruitment and activation are suppressed in the plaque. (**B**) LXR deficiency in myeloid cells induces cholesterol accumulation, impairs TREM2′s expression and its downstream signaling, and then switches foamy macrophages to the inflammatory phenotype. Activated foamy and inflammatory macrophages increase neutrophil extracellular traps (NETosis), necrotic core formation, and atherosclerosis progression [179]. Red arrows, up; blue arrows, down.

The roles of LXRs in immune cells throughout the pathogenesis of atherosclerosis have been investigated using the BMT of LXR-deficient cells in an atherosclerotic mouse model. The transplantation of bone marrow cells from LXRα/β-deficient mice exacerbated atherosclerosis in both Apo E-deficient and LDLR-deficient mice [177] (Table 2), and the atheroprotective effects of the LXR agonist T0901317 were abolished in LXRα/β-knockout cell-transplanted mice [178], demonstrating the atheroprotective functions of LXRs in hematopoietic cells. The BMT of myeloid-selective LXRα/β-deficient cells in LDLR-deficient mice accelerated atherosclerosis, and they were associated with enhanced myeloid cell recruitment, proliferation, and activation [179] (Figure 3). scRNA-seq analyses of CD45-positive immune cells in aortic plaques showed that non-inflammatory TREM2-positive foamy macrophages are increased via LXR deletion, where these cells abnormally differentiated into inflammatory phenotypes. Increased inflammation suppressed TREM2′s expression and its phagocytic activity. The expression of the *Trem2* gene is regulated by LXRs in Kupffer cells [38] but not in bone-marrow-derived macrophages [179]. These findings indicate the functional roles of LXRs in TREM2-positive macrophages and the cell-type selective role of LXRs regarding *Trem2* expression. The deletion of TREM2 impaired LXR-mediated cholesterol efflux and endoplasmic reticulum stress pathways in macrophages [30]. These findings suggest that LXRs and TREM2 play reciprocal roles with respect to macrophage functions. Myeloid cell LXR deletion enhanced neutrophil infiltration and NETosis in atherosclerotic lesions [179]. LXRs are involved in neutrophils’ phagocytic activity and clearance, regulating IL-23/IL-17/granulocyte-colony-stimulating-factor granulopoietic cytokine signaling [107]. LXR activation in macrophages suppressed genes involved in neutrophil migration [86]. Cholesterol accumulation in macrophages stimulated IL-1β production via inflammasome activation, consequently inducing NETosis [138]. However, the direct effects of neutrophil LXRs on the induction of NETosis in atherosclerotic plaques remain unclear.

In addition to atherogenesis, LXRs are also involved in the regression stage of atherosclerosis. The C-C chemokine receptor (CCR)7 is required for the egress of CD68-positive monocytes/macrophages from an atherosclerotic lesion during regression. LXR activation promoted CCR7 expression and plaque regression in Apo E-null mice [180] (Table 2). The transfer of aortic arches isolated from LXRα-deficient or LXRβ-deficient mice into wild-type mice impaired the elimination of CD68-positive cells from the plaque. Mechanistically, the expression of the *CCR7* gene is directly regulated by LXRs in human and mouse dendritic cells [180]. LXRα activation also induces the expression of mouse macrophage arginase-1, which is involved in plaque stability and tissue repair, in regression stages through the expression and activation of interferon regulatory factor 8 [181]. Cyclodextrin, a compound that increases lipid solubility, has been shown to suppress atherogenesis and promote the regression of established atherosclerosis [182]. Cholesterol crystals are dissolved by cyclodextrin, resulting in oxysterol production and LXR-dependent macrophage reprogramming. Therefore, LXRs play central roles in regulating both the progression and regression of atherosclerosis in foamy macrophages.

LXRs are expressed in other immune cells, such as lymphocytes, neutrophils, and dendritic cells. Thus, their roles in the pathogenesis of atherosclerosis still require full elucidation. Further studies are needed to elucidate the cell-selective functions of LXRs in various immune cells.

**Table 2:** Effects of LXR deletion in animal models of atherosclerosis.

Mice	Model	LXR	Diet	Treatment	Findings	Ref.
Apo E(-/-); LXRα(-/-)	Progression	Genetic deletion	WTD (14 or 34 weeks)	GW3965 (20 mpk)	Cholesterol accumulation in tissues Lesion size↑ GW3965 administration reduces lesion size	[172]
LDLR(-/-); LXRα(-/-) LDLR(-/-); LXRβ(-/-)	Progression	Genetic deletion	WTD (20 weeks)	T0901317 (10→3 mpk)	Lesion size↑ [LXRα(-/-)] Lesion size→ [LXRβ(-/-)] T0901317 administration reduces lesion size [LXRα(-/-) and LXRβ(-/-)]	[173]
Apo E(-/-); LXRα(-/-) Apo E(-/-); LXRβ(-/-)	Progression	Genetic deletion	WTD (15 weeks)		Lesion size↑ [LXRα(-/-)] Lesion size→ [LXRβ(-/-)]	[174]
LDLR(-/-); Liver-specific LXRα(-/-)	Progression	Genetic deletion (liver)	WTD (20 weeks)		Cholesterol excretion↓ Lesion size↑	[175]
Apo E(-/-) and LDLR(-/-)	Progression	BMT [donor: LXRα(-/-); LXRβ(-/-)]	WTD (8 or 16 weeks)	T0901317 (10 mpk)	Lesion size↑ No effect on T0901317 treatment	[177,178]
LDLR(-/-)	Progression	BMT [donor: myeloid-specific LXRα(-/-); LXRβ(-/-)]	WTD (10 weeks)		Lesion size↑ Foamy macrophage activation↑ NETosis↑	[179]
Apo E(-/-)	Regression	BMT [donor: Apo E(-/-); LXRα(-/-) or Apo E(-/-); LXRβ(-/-)]	WTD (16 weeks)		Aortic arch transplantation to wild-type mice impairs plaque regression CCL7 expression↓	[180]

WTD, Western diet high in fat and cholesterol; BMT, bone marrow transplantation. Arrows indicate the following: ↑, increase; ↓, decrease; →, no change.

## 7. Post-Translational Modification of LXR in Atherosclerosis

LXRs are known to be post-translationally modified via phosphorylation [183,184], ubiquitination [185,186], acetylation [187], O-linked β-N-acetylglucosamination [188], and SUMOylation [81,82,83].

Human LXRα is phosphorylated at serine 198, and LXRβ is phosphorylated at serine 196 [183]. The transactivation activity of the LXRα S198A mutant was comparable to that of LXRα WT. The phosphorylation of LXRα S198 inhibited the expression of C-C chemokine ligand (CCL) 24 in macrophages [184]. Mouse LXRα was phosphorylated at S196 (corresponding to S198 in human LXRα) in atherosclerotic plaques during the progression stages, whereas it was not phosphorylated in the regression stages and was associated with CCL7 expression, suggesting that the phosphorylation of LXRα S196 is important for the regression of plaques through CCL7 expression [189]. The introduction of myeloid cell-specific LXRα S196A in LDLR-deficient mice promoted atherosclerosis, which is associated with macrophage proliferation, and upregulated the expression of genes such as transcription factor forkhead box M1 [190] (Table 3). In contrast, LXRα S196A reduced the size of the necrotic core and promoted efferocytosis. BMT from LXRα S196A mice decreased atherosclerosis by reducing inflammatory monocyte migration, macrophage proliferation, and apoptosis [191]; however, the observed discrepancy may be due to the experimental conditions. Further studies are needed to clarify the role of LXRα phosphorylation in atherosclerosis.

**Table 3:** Effects of LXR post-translational modification in animal models of atherosclerosis.

Mice	Model	LXR	Diet	Findings	Ref.
LDLR(-/-); Myeloid specific LXRα S196A knock-in	Progression	Genetic modification (disruption of LXRα phosphorylation)	WTD (12 weeks)	Lesion size↑ Macrophage proliferation↑ Necrotic core↓ Efferocytosis↓	[190]
LDLR(-/-)	Progression	BMT [LXRα(-/-) S196A] (disruption of LXRα phosphorylation)	WTD (16 weeks)	Macrophage proliferation↓ Monocyte recruitment↓ Apoptosis↓	[191]
LDLR(-/-); TTC39B(-/-)	Progression	Endogenous LXRα protein stability↑	WTD (16 weeks)	Lesion size↓ Plaque severity↓ HDL-C↑, LDL-C↓	[185]

WTD, Western diet high in fat and cholesterol; BMT, bone marrow transplantation. Arrows indicate the following: ↑, increase; ↓, decrease.

The stability and degradation of LXRα are regulated by tetratricopeptide repeat domain protein 39B (TTC39B) [185]. A single-nucleotide polymorphism of *TTC39B* has been associated with plasma HDL-cholesterol levels and coronary artery disease [192]. TTC39B acts as an E3 ligase for LXRα in hepatocytes. TTC39B deletions in LDLR-deficient mice stabilized the LXRα protein, increased plasma HDL-cholesterol levels, and provided protection against atherosclerosis. The degradation of LXRα was also induced by another E3-ligase protein complex containing breast and ovarian cancer susceptibility 1-associated RING domain 1, and LXR ligand binding increased LXRα protein levels by suppressing their degradation [186]. Thus, LXR activities are regulated by both ligand binding and post-translational modification. The roles of the acetylation, O-linked β-N-acetylglucosamination, and SUMOylation of LXRs in the pathogenesis of atherosclerosis remain unclear.

## 8. Effects of Other Nuclear Receptors on Atherosclerosis

Farnesoid X receptor (FXR) and peroxisome proliferator-activated receptors (PPARs) are other nuclear receptors that regulate lipid metabolism and inflammatory responses. These receptors belong to the nuclear receptor 1 subfamily, form heterodimers with RXR-like LXRs, and influence atherosclerosis progression cooperatively with LXRs.

### 8.1. FXR

FXR (NR1H4) is activated by bile acids such as chenodeoxycholic acid [193], and it regulates cholesterol and bile acid metabolism. FXR activation or deletion influences atherosclerosis progression in animal models. Pharmacological FXR activation with the synthetic ligand WAY362450 ameliorates atherosclerosis by reducing non-HDL levels and the expression of bile acid synthetic genes [194]. Another ligand, INT-747, also improves plasma lipoprotein profiles, decreases the expression of inflammatory genes, and suppresses atherosclerosis [195]. Whole-body FXR deletions in LDLR-null mice increase plasma VLDL and LDL cholesterol levels and decrease HDL levels, resulting in atherosclerosis progression [196]. These findings suggest that FXR acts as an antiatherogenic factor. On the other hand, FXR deletion in Apo E-knockout or LDLR-knockout mice ameliorates atherosclerosis by reducing plasma LDL levels and decreasing the expression of the scavenger receptor CD36 in macrophages [197,198]. The intestine-selective deletion of FXR also suppresses atherosclerosis along with decreasing serum ceramide and cholesterol levels by reducing intestinal ceramide synthesis [199]. Therefore, the effect of FXR on atherosclerosis remains controversial. The discrepancies may be due to experimental conditions, and further studies are needed to clarify the detailed mechanisms of FXR function in atherosclerosis pathogenesis.

### 8.2. PPARs

PPARs consist of the following three isoforms: PPARα (NR1C1), PPARδ/β (NR1C2), and PPARγ (NR1C3). PPARα is highly expressed in the liver and is activated by fibrates; moreover, it regulates the expression of genes involved in fatty acid transport, oxidation, and esterification [200]. PPARγ is mainly present in the adipose tissue; it is activated by thiazolidinediones and regulates genes involved in fatty acid transportation, lipid synthesis, adipogenesis, energy storage, thermogenesis, and glucose metabolism. PPARδ/β is ubiquitously expressed and regulates fatty acid oxidation and glucose metabolism. Thus, PPARs have been suggested to influence the pathogenesis of atherosclerosis by regulating lipid and energy metabolism. The effects of the activation or deletion of PPARα [201], PPARδ/β [202], and PPARγ [203], especially in macrophages, on atherosclerosis have been studied in animal models. The activation of these receptors prevents atherosclerosis by regulating cholesterol metabolism and inflammatory responses. Importantly, PPARγ increases ABCA1 expression and promotes cholesterol efflux in macrophages through the induction of LXRα expression [203]. Clinical trials with pan-PPAR or selective-PPAR modulators have been performed in patients with type 2 diabetes, hyperlipidemia, and atherosclerosis [204]. These studies revealed the beneficial effects of PPAR ligands in improving lipoprotein profiles, enhancing endothelial cell function, and reducing inflammation.

## 9. Concluding Remarks

LXRs play important roles in regulating lipid metabolism and immunity in leukocytes, especially macrophages, in the context of atherosclerosis. LXR activation promotes cholesterol efflux and may suppress inflammatory responses in macrophages, thus exerting an atheroprotective effect. The deletion of LXRs in myeloid cells converts non-inflammatory foamy macrophages to the inflammatory phenotype, exacerbating the progression of atherosclerosis. Therefore, immune cell LXRs are very important in providing protection against atherosclerosis, while LXRs in metabolic cells, such as hepatocytes and intestinal epithelial cells, regulate whole-body lipid metabolism. Several loss-of-function LXRα mutations have recently been identified in the human UK Biobank database [205]. These mutations are positively associated with liver injury in both human and model animals [205,206]. The effects of these mutations on immune responses or cardiovascular disease are yet to be elucidated. In summary, LXRs may be considered a potent therapeutic target for the treatment of hypercholesterolemia and atherosclerosis. As LXR agonists can have adverse effects, such as neuropsychiatric dysfunction and hypertriglyceridemia, the development of function-selective or cell-type-selective LXR ligands holds promise for clinical applications.

## Data Availability

Not applicable.

## Abbreviations

ABC	ATP-binding cassette
Apo E	Apolipoprotein E
BMT	Bone marrow transplantation
CCL	C-C chemokine ligand
CCR	C-C chemokine receptor
CITE-seq	Cellular indexing of transcriptomes and epitopes by sequencing
CH25H	Cholesterol 25-hydroxylase
CYP7A1	Cholesterol 7α-hydroxylase
cDC	Conventional dendritic cell
DC	Dendritic cell
DHCR24	24-dehydrocholesterol reductase
FXR	Farnesoid X receptor
FAS	Fatty acid synthase
HDL	High-density lipoprotein
iNOS	Inducible NO synthase
IFN	Interferon
IL	Interleukin
LBP	Lipopolysaccharide-binding protein
LXR	Liver X receptor
LDL	Low-density lipoprotein
LDLR	Low-density lipoprotein receptor
M-CSF	Macrophage colony-stimulating factor
MHC	Major histocompatibility complex
NK	Natural killer
NET	Neutrophil extracellular trap
NADPH	Nicotinamide adenine dinucleotide phosphate hydrogen
NLRP3	NLR family pyrin domain containing 3
pDC	Plasmacytoid dendritic cell
PAD	Peptidylarginine deiminase
PPAR	Peroxisome proliferator-activated receptor
rHDL	Reconstituted high-density lipoprotein
RAR	Retinoic acid receptor
SCD1	Stearoyl-CoA desaturase-1
scRNA-seq	Single-cell RNA sequencing
SREBP1c	Sterol regulatory element-binding protein-1c
SUMO	Small ubiquitin-like modifier
TLR	Toll-like receptor
TTC39B	Tetratricopeptide repeat domain protein 39B
TGM2	Transglutaminase 2
TREM2	Triggering receptor expressed on myeloid cells 2
